# Crystal structure and Hirshfeld surface analysis of 10-hy­droxy-2-(4-meth­oxy­phen­yl)-3-oxo-2,3,3a,4,10,10a-hexa­hydro-1*H*-9-thia-2-aza­cyclo­penta­[*b*]fluorene-4-carb­oxy­lic acid dimethyl sulfoxide-*d*_6_ monosolvate

**DOI:** 10.1107/S2056989023009635

**Published:** 2023-11-10

**Authors:** Gunay Z. Mammadova, Elizaveta D. Yakovleva, Pavel P. Erokhin, Mikhail S. Grigoriev, Zeliha Atioğlu, Asmet N. Azizova, Mehmet Akkurt, Ajaya Bhattarai

**Affiliations:** a Organic Chemistry Department, Baku State University, Z. Xalilov Str. 23, Az 1148 Baku, Azerbaijan; b Peoples’ Friendship University of Russia (RUDN University), 6 Miklukho-Maklaya St., Moscow, 117198, Russian Federation; c Frumkin Institute of Physical Chemistry and Electrochemistry, Russian Academy of Sciences, Leninskiy prospect 31-4, Moscow 119071, Russian Federation; d Department of Aircraft Electrics and Electronics, School of Applied Sciences, Cappadocia University, Mustafapaşa, 50420 Ürgüp, Nevşehir, Türkiye; e Department of Synthesis of Biologically Active Compounds, Scientific Research Center, Azerbaijan Medical University, Samed Vurgun St. 167, Az 1022 Baku, Azerbaijan; f Department of Physics, Faculty of Sciences, Erciyes University, 38039 Kayseri, Türkiye; g Department of Chemistry, M.M.A.M.C (Tribhuvan University), Biratnagar, Nepal; University of Neuchâtel, Switzerland

**Keywords:** crystal structure, disorder, dimer, hydrogen bonds, Hirshfeld surface analysis

## Abstract

In the title compound, C_22_H_19_NO_5_S·C_2_D_6_OS, the central six-membered ring has a slightly distorted boat conformation, while the fused pyrrolidine ring adopts an envelope conformation. These conformations are stabilized by O—H⋯O hydrogen bonds between the main compound and solvent mol­ecules. In addition, intra­molecular C—H⋯O hydrogen bonds in the main mol­ecule form two *S*(6) rings. Mol­ecules are connected by pairs of inter­molecular C—H⋯O hydrogen bonds, forming dimers with a *R* ^2^_2_(8) motif. These dimers form a three-dimensional network through O—H⋯O, O—H⋯S and C—H⋯O hydrogen bonds with each other directly and through solvent mol­ecules. In addition, weak π–π stacking inter­actions [centroid-to-centroid distances = 3.9937 (10) and 3.9936 (10) Å, slippages of 2.034 and 1.681 Å] are observed. The inter­molecular contacts were qu­anti­fied using Hirshfeld surface analysis and two-dimensional fingerprint plots, revealing the relative contributions of the contacts to the crystal packing to be H⋯H 41.7%, O⋯H/H⋯O 27.7%, C⋯H/H⋯C 17.0%, and S⋯H/H⋯S 7.5%.

## Abstract 2

In the crystal, mol­ecules are connected by pairs of inter­molecular C—H⋯O hydrogen bonds, forming dimers with 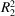 (8) motifs. These dimers form a three-dimensional network through O—H⋯O, O—H⋯S and C—H⋯O hydrogen bonds with each other directly and through solvent mol­ecules.

## 1. Chemical context

Inter­molecular non-covalent inter­actions play a critical role in determining the crystal packing and orientation of organic and coordination compounds, leading to significant changes in their properties and actions (Gurbanov *et al.*, 2018 ▸, 2020 ▸; Kopylovich *et al.*, 2011*a* ▸,*b* ▸,*c* ▸; Mahmoudi *et al.*, 2019 ▸, 2021 ▸; Mahmudov *et al.*, 2013 ▸). In fact, various types of non-covalent bond donors and acceptors determine the supra­molecular packing of heterocyclic and coordination compounds, which is a fundamental mol­ecular descriptor for predicting the oral bioavailability as well as biocatalytic activity of small drug candidates (Abdelhamid *et al.*, 2011 ▸; Akbari Afkhami *et al.*, 2017 ▸; Khalilov *et al.*, 2021 ▸; Safavora *et al.*, 2019 ▸). This work is a continuation of studies of properties of vinyl­arene systems, previously obtained by the tandem acyl­ation/[4 + 2]-cyclo­addition between 3-(ar­yl)allyl­amines and maleic anhydrides as an example of an IMDAV (Intra Mol­ecular Diels–Alder Vinyl­arene) reaction. The IMDAV reaction is a useful tool for the one-step synthesis of benzo­furans, indoles and benzo­thio­phenes annalated with other carbo- or heterocycles (Horak *et al.*, 2015 ▸, 2017 ▸; Krishna *et al.*, 2022 ▸; Nadirova *et al.*, 2020 ▸; Zubkov *et al.*, 2016 ▸).

**Figure 1:**
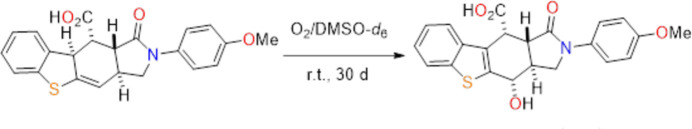
Synthesis of 10-hy­droxy-2-(4-meth­oxy-phen­yl)-3-oxo-2,3,3a,4,10,10*a*-hexa­hydro-1*H*-9-thia-2-aza-cyclo­penta­[*b*]fluorene-4-carb­oxy­lic acid.

We report here the first case of a spontaneous oxidation reaction of an IMDAV adduct (Fig. 1 ▸) in air in DMSO at room temperature. Presumably, the DMSO acts as a mild oxidant, as it is observed in a number of other oxidation reactions – Pfitzner-Moffatt, Corey–Kim, Swern, and Kornblum oxidation (Epstein *et al.*, 1967 ▸). The slow oxidation of (3a*RS*,9b*RS*,10*RS*,10a*RS*)-2-(4-meth­oxy­phen­yl)-1-oxo-2,3,3a,4,10,10a-hexa­hydro-1*H*-benzo[4,5]thieno[2,3-*f*]iso­indole-10-carb­oxy­lic acid occurs under stirring of the solution in DMSO-*d*_6_ for a month. The title compound was isolated in 67% yield after a standard treatment of the reaction mixture. It should be noted that in this case, the reaction does not stop at the formation of an alcohol, but leads to the formation of an aromatic product as a result of proton migration. 
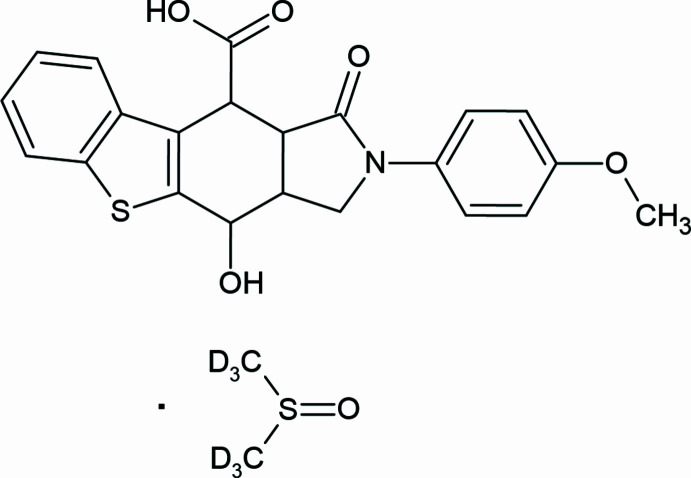


## 2. Structural commentary

**Figure 2:**
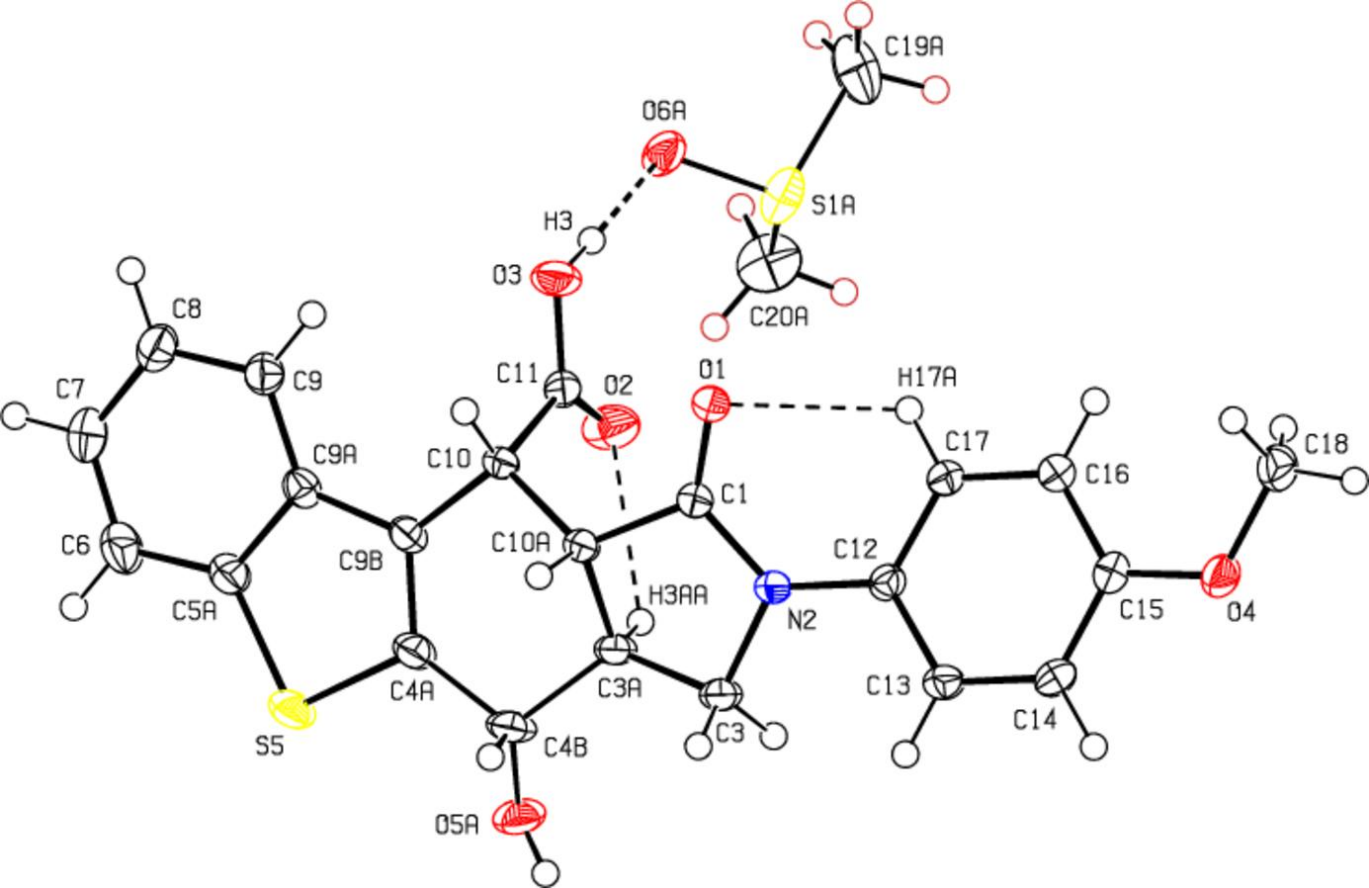
The mol­ecular structure of the title compound, with atom labeling. Displacement ellipsoids are drawn at the 50% probability level. Only the major component of the disordered DMSO mol­ecule is shown.

In the title compound (Fig. 2 ▸), the central six-membered ring (C3*A*/C4*B*/C4*A*/C9*B*/C10/C10*A*) has a slightly distorted boat conformation, with puckering parameters (Cremer & Pople, 1975 ▸) of *Q*_T_ = 0.5290 (17) Å, θ = 129.87 (18)° and φ = 156.7 (2)°. The fused pyrrolidine ring (N2/C1/C10*A*/C3*A*/C3) adopts an envelope conformation with the C3*A* atom as the flap [the puckering parameters are *Q*(2) = 0.3523 (17) Å and φ(2) = 290.0 (3)°], while the fused thio­phene ring (S5/C4*A*/C9*B*/C9*A*/C5*A*) is essentially planar (r.m.s. deviation = 0.002 Å). The mol­ecular conformation is stabilized by an O—H⋯O hydrogen bond (O3—H3⋯O6*A*) between the main compound and solvent mol­ecules, as well as two intra­molecular C—H⋯O hydrogen bonds (C17—H17*A*⋯O1 and C3*A*—H3*AA*⋯O2) in the main mol­ecule, which form *S*(6) rings (O1/C1/N2/C12/C17/H17*A* and O2/C11/C10/C10*A*/C3*A*/H3*AA*; Table 1 ▸; Fig. 2 ▸; Bernstein *et al.*, 1995 ▸). All bond lengths and angles in the main compound are comparable to those of the analogous compound ethyl 2-methyl-5,8-dioxo-6-phenyl-4a,5,6,7,7a,8-hexa­hydro-4*H*-furo[2,3-*f*]iso­indole-4-carboxyl­ate (CSD refcode OJIPUV; Zaytsev *et al.*, 2021 ▸).

## 3. Supra­molecular features and Hirshfeld surface analysis

**Figure 3:**
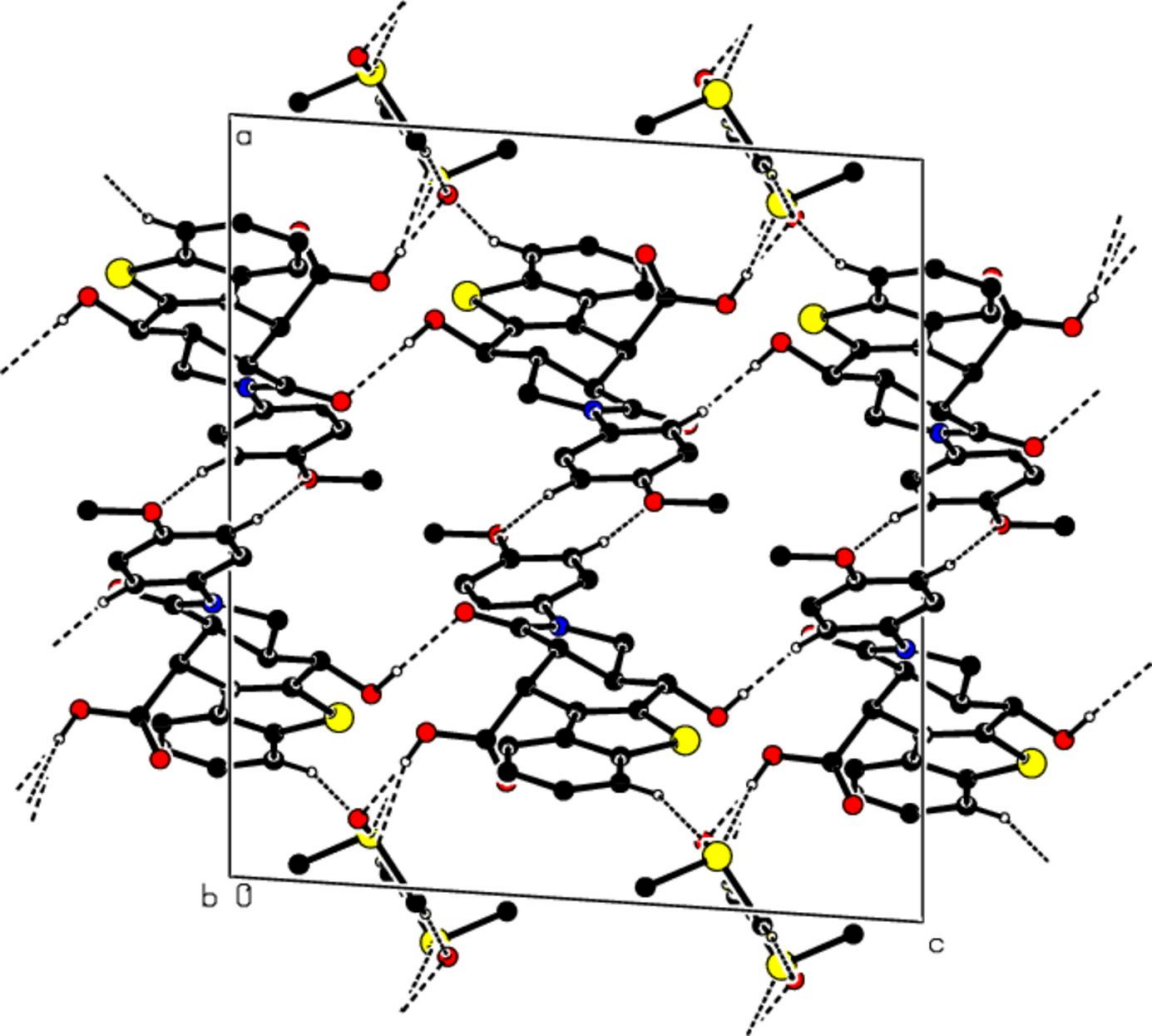
A view along the *b-*axis of the crystal packing of the title compound. The O—H⋯O, O—H⋯S and C—H⋯O hydrogen bonds are shown as dashed lines. Only the major component of the disordered DMSO mol­ecule is shown.

In the crystal structure of the title compound, mol­ecules are connected by pairs of inter­molecular C—H⋯O hydrogen bonds, forming dimers with an 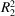 (8) motif (Table 1 ▸, Fig. 3 ▸). These dimers form a three-dimensional network through O—H⋯O, O—H⋯S and C—H⋯O hydrogen bonds, directly with each other and through solvent mol­ecules (Table 1 ▸). In addition, weak π–π stacking inter­actions are observed [*Cg*5⋯*Cg*6(*x*, 1 + *y*, *z*) = 3.9937 (10) Å with slippage of 2.034 Å and *Cg*6⋯*Cg*5(*x*, −1 + *y*, *z*) = 3.9936 (10) Å with slippage of 1.681 Å; *Cg*5 and *Cg*6 are the centroids of the C5*A*/C6/C7/C8/C9/C9*A* and C12–C17 benzene rings, respectively].

Hirshfeld surfaces and their associated two-dimensional fingerprint plots were used to qu­antify the various inter­molecular inter­actions, and were generated using *Crystal Explorer* 17.5 (Spackman *et al.*, 2021 ▸). The 3D *d*_norm_ surfaces are plotted over a fixed color scale of −0.7960 (red) and 1.2965 (blue) a.u.

**Figure 4:**
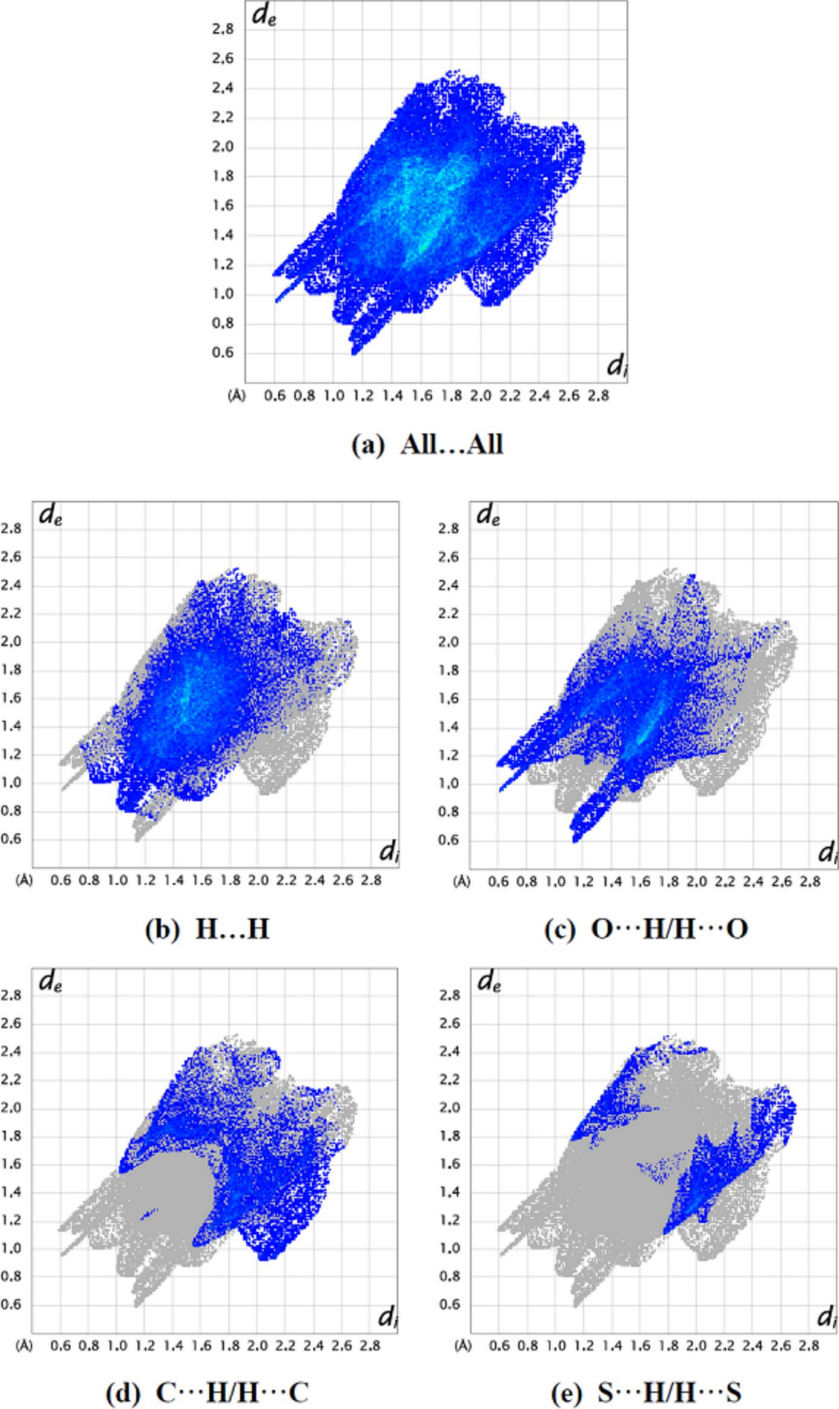
The two-dimensional fingerprint plots of the title compound, showing (*a*) all inter­actions, and delineated into (*b*) H⋯H, (*c*) O⋯H/H⋯O, (*d*) C⋯H/H⋯C and (*e*) S⋯H/H⋯S inter­actions. [*d*_e_ and *d*_i_ represent the distances from a point on the Hirshfeld surface to the nearest atoms outside (external) and inside (inter­nal) the surface, respectively].

Two-dimensional fingerprint plots together with their percentage contributions are shown in Fig. 4 ▸. The crystal packing is dominated by H⋯H contacts, representing van der Waals inter­actions (41.7% contribution to the overall surface), followed by O⋯H/H⋯O, C⋯H/H⋯C and S⋯H/H⋯S inter­actions, which contribute to 27.7%, 17.0% and 7.5%, respectively. The other contacts (C⋯C 4.2%, N⋯C/C⋯N 1.3%, O⋯O 0.7%, N⋯H/H⋯N 0.1% and S⋯C/C⋯S 0.1%) only make a minor contribution to the crystal packing.

## 4. Database survey

A search of the Cambridge Crystallographic Database (CSD version 5.40, update of September 2019; Groom *et al.*, 2016 ▸) yielded six entries closely related to the title compound, *viz*. OJIPUV (Zaytsev *et al.*, 2021 ▸), JOGYIP (Zhou *et al.*, 2014 ▸), LESXIS (Horak *et al.*, 2013 ▸), QAFSUO (Zubkov *et al.*, 2016 ▸), QAFTAV (Zubkov *et al.*, 2016 ▸) and QUKPAP (Horak *et al.*, 2015 ▸).

In OJIPUV and JOGYIP, space group *P* 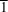 , mol­ecules are bonded by inter­molecular C—H⋯O hydrogen bonds, C—H⋯·π inter­actions, and π–π stacking inter­actions, forming three-dimensional networks. In the crystal of LESXIS (*Pbca*), which contains two similar mol­ecules per asymmetric unit, O—H⋯O hydrogen bonds connect the mol­ecules into chains parallel to the *b*-axis. There are also weak C—H⋯π inter­actions in the crystal. In the crystal structures of QAFSUO (*P*2_1_/*c*) and QAFTAV (*P*2_1_/*n*), the three-dimensional packings are stabilized by O—H⋯O hydrogen bonds, C—H⋯O contacts and C—H⋯π inter­actions. The asymmetric unit of QUKPAP (*P*2_1_/*c*) comprises two similar mol­ecules, *A* and *B*, of the same chirality. The only considerable difference concerns the conformation of the allyl group. The carboxyl hydrogen atoms are involved in strong hydrogen bonds with the carbonyl atoms of neighboring mol­ecules, giving rise to (*A*⋯*B*⋯)_*n*_ chains.

In the six structures, the different groups bonded to the central twelve-membered ring systems account for the distinct inter­molecular inter­actions in the crystals.

## 5. Synthesis and crystallization

A solution of (3a*RS*,9b*RS*,10*RS*,10a*RS*)-2-(4-meth­oxy­phen­yl)-1-oxo-2,3,3a,4,10,10a-hexa­hydro-1*H*-benzo[4,5]thieno[2,3-*f*]iso­indole-10-carb­oxy­lic acid (30.0 mg, 0.08 mmol) in 0.5 ml of DMSO-*d*_6_ was stirred for 30 days in an open flask. The reaction mixture was concentrated, diluted with EtOH (0.5 mL), and the solid was filtered, washed with Et_2_O (3 × 1 mL), and air dried. The title compound was obtained as a colorless powder, yield 67%, 25.2 mg; m.p. > 523 K (with decomp.). IR (KBr), *ν* (cm^−1^): 1722 (CO_2_), 1644 (N—C=O), 1514. ^1^H NMR (700.2 MHz, DMSO-*d_6_*): *δ* (*J*, Hz) *there are no OH peak*s 12.78 (*s*, 1H, CO_2_H), 8.04 (*d*, *J* = 7.6, 1H, H Ar), 7.92 (*d*, *J* = 7.6, 1H, H Ar), 7.59 (*d*, *J* = 9.1, 2H, H Ar), 7.42 (*t*, *J* = 7.6, 1H, H Ar), 7.34 (*t*, *J* = 7.6, 1H, H Ar), 6.97 (*d*, *J* = 9.1, 2H, H Ar), 2.47–2.44 (*m*, 1H, H-4) 4.28 (*d*, *J* = 4.8, 1H, H-10), 4.00 (*t*, *J* = 8.7, 1H, H-3A), 3.75 (*s*, 3H, CH_3_), 3.73 (*t*, *J* = 8.7, 1H, H-3B), 3.40–3.37 (*m*, 1H, H-3a), 3.21 (*dd*, *J* = 16.0, 4.8, 1H, H-10a). ^13^C{^1^H} NMR (176.1 MHz, DMSO-*d_6_*): *δ* 172.7, 172.2, 156.1, 139.6, 138.8, 138.4, 133.5, 126.7, 124.7, 124.6, 122.9, 122.7, 121.1 (2C), 114.3 (2C), 68.2, 55.7, 52.2, 47.8, 40.5, 32.7. MS (ESI) *m/z*: [M + H]^+^ 494. Elemental analysis calculated (%) for C_22_H_19_NO_5_S·C_2_D_6_OS: C 58.40, H 6.33, N 2.84, S 12.99; found: C 58.13, H 6.47, N 3.07, S 13.20.

## 6. Refinement

Crystal data, data collection and structure refinement details are summarized in Table 2 ▸. The H atoms of the OH groups were placed in geometrically idealized positions and constrained to ride on their parent atoms, with O—H = 0.84 Å and *U*_iso_(H) = 1.5*U*_eq_(O). H atoms bound to C atoms were placed in geometrically idealized positions and constrained to ride on their parent atoms, with C—H = 0.95–1.00 Å and *U*_iso_(H) = 1.2 or 1.5*U*_eq_(C). The dimethyl sulfoxide solvent mol­ecule exhibits disorder at two positions in the ratio 0.8903 (18):0.1097 (18). All the methyl hydrogen atoms of the solvent mol­ecule were assigned as deuterium and refined. The C4*B* and C4*C* atoms of the two parts of the disordered solvent mol­ecule were refined using EADP and EXYZ commands, and other similar bond lengths of the disordered solvent mol­ecule were refined using SADI.

## Supplementary Material

Crystal structure: contains datablock(s) I. DOI: 10.1107/S2056989023009635/tx2077sup1.cif

Structure factors: contains datablock(s) I. DOI: 10.1107/S2056989023009635/tx2077Isup2.hkl

CCDC reference: 2305649

Additional supporting information:  crystallographic information; 3D view; checkCIF report

## Figures and Tables

**Table 1:** Hydrogen-bond geometry (Å, °)

*D*—H⋯*A*	*D*—H	H⋯*A*	*D*⋯*A*	*D*—H⋯*A*
O3—H3⋯S1*A*	0.84	2.72	3.4846 (15)	152
O3—H3⋯O6*A*	0.84	1.72	2.563 (2)	176
O3—H3⋯O6*B*	0.84	2.06	2.852 (16)	158
O5*A*—H5*A*⋯O1^i^	0.84	1.96	2.763 (2)	160
C3*A*—H3*AA*⋯O2	1.00	2.49	3.202 (2)	127
C3—H3*A*⋯O5*B*	0.99	2.54	2.887 (4)	100
C6—H6*A*⋯O6*A* ^ii^	0.95	2.48	3.307 (3)	145
C14—H14*A*⋯O4^iii^	0.95	2.54	3.445 (2)	159
C17—H17*A*⋯O1	0.95	2.33	2.868 (2)	116
C17—H17*A*⋯O5*B* ^iv^	0.95	2.46	3.289 (4)	146
C18—H18*C*⋯O5*B* ^v^	0.98	2.43	2.922 (4)	110
C20*A*—D20*A*⋯O6*A* ^vi^	0.98	2.46	3.434 (3)	173
C20*A*—D20*A*⋯O6*B* ^vi^	0.98	1.87	2.839 (13)	168

Symmetry codes: (i) 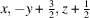 ; (ii) 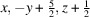 ; (iii) 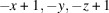 ; (iv) 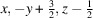 ; (v) 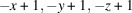 ; (vi) 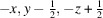 .

**Table 2:** Experimental details

Crystal data	
Chemical formula	C_22_H_19_NO_5_S·C_2_D_6_OS
*M*_r_	493.61
Crystal system, space group	Monoclinic, *P*2_1_/*c*
Temperature (K)	100
*a*, *b*, *c* (Å)	16.3178 (4), 9.2747 (2), 14.8720 (4)
β (°)	93.771 (1)
*V* (Å^3^)	2245.89 (10)
*Z*	4
Radiation type	Mo *K*α
μ (mm^−1^)	0.28
Crystal size (mm)	0.40 × 0.28 × 0.22

Data collection	
Diffractometer	Bruker Kappa APEXII area-detector diffractometer
Absorption correction	Multi-scan (*SADABS*; Krause *et al.*, 2015 ▸)
*T*_min_, *T*_max_	0.847, 0.941
No. of measured, independent and observed [*I* > 2σ(*I*)] reflections	69078, 6531, 5693
*R*_int_	0.032
(sin θ/λ)_max_ (Å^−1^)	0.703

Refinement	
*R*[*F* ^2^ > 2σ(*F* ^2^)], *wR*(*F* ^2^), *S*	0.049, 0.129, 1.12
No. of reflections	6531
No. of parameters	324
No. of restraints	15
H-atom treatment	H-atom parameters constrained
Δρ_max_, Δρ_min_ (e Å^−3^)	0.54, −0.47

Computer programs: *APEX4* and *SAINT* (Bruker, 2008 ▸), *SHELXT2016/6* (Sheldrick, 2015*a* ▸), *SHELXL2016/6* (Sheldrick, 2015*b* ▸, *ORTEP-3 for Windows* (Farrugia, 2012 ▸) and *PLATON* (Spek, 2020 ▸).

## supplementary crystallographic information

### Crystal data

**Table:**

C_22_H_19_NO_5_S·C_2_D_6_OS	*F*(000) = 1024
*M**_r_* = 493.61	*D*_x_ = 1.460 Mg m^−^^3^
Monoclinic, *P*2_1_/*c*	Mo *K*α radiation, λ = 0.71073 Å
*a* = 16.3178 (4) Å	Cell parameters from 9765 reflections
*b* = 9.2747 (2) Å	θ = 2.6–30.1°
*c* = 14.8720 (4) Å	µ = 0.28 mm^−^^1^
β = 93.771 (1)°	*T* = 100 K
*V* = 2245.89 (10) Å^3^	Fragment, colourless
*Z* = 4	0.40 × 0.28 × 0.22 mm

### Data collection

**Table:**

Bruker Kappa APEXII area-detector diffractometer	5693 reflections with *I* > 2σ(*I*)
φ and ω scans	*R*_int_ = 0.032
Absorption correction: multi-scan (SADABS; Krause *et al.*, 2015)	θ_max_ = 30.0°, θ_min_ = 4.2°
*T*_min_ = 0.847, *T*_max_ = 0.941	*h* = −22→22
69078 measured reflections	*k* = −13→13
6531 independent reflections	*l* = −20→20

### Refinement

**Table:**

Refinement on *F*^2^	15 restraints
Least-squares matrix: full	Hydrogen site location: inferred from neighbouring sites
*R*[*F*^2^ > 2σ(*F*^2^)] = 0.049	H-atom parameters constrained
*wR*(*F*^2^) = 0.129	*w* = 1/[σ^2^(*F*_o_^2^) + (0.0518*P*)^2^ + 1.8241*P*] where *P* = (*F*_o_^2^ + 2*F*_c_^2^)/3
*S* = 1.12	(Δ/σ)_max_ = 0.001
6531 reflections	Δρ_max_ = 0.54 e Å^−^^3^
324 parameters	Δρ_min_ = −0.47 e Å^−^^3^

### Special details

**Table:**

Geometry. All esds (except the esd in the dihedral angle between two l.s. planes) are estimated using the full covariance matrix. The cell esds are taken into account individually in the estimation of esds in distances, angles and torsion angles; correlations between esds in cell parameters are only used when they are defined by crystal symmetry. An approximate (isotropic) treatment of cell esds is used for estimating esds involving l.s. planes.

### Fractional atomic coordinates and isotropic or equivalent isotropic displacement parameters (Å^2^)

**Table:**

	*x*	*y*	*z*	*U*_iso_*/*U*_eq_	Occ. (<1)
S1A	0.06873 (3)	0.67027 (5)	0.20351 (4)	0.03201 (16)	0.8903 (18)
S1B	−0.0094 (3)	0.7350 (5)	0.2022 (3)	0.0386 (13)*	0.1097 (18)
S5	0.21789 (3)	1.16195 (5)	0.65744 (3)	0.03355 (13)	
O1	0.36715 (8)	0.72156 (13)	0.33936 (8)	0.0253 (3)	
O2	0.14801 (9)	0.83115 (17)	0.39946 (10)	0.0367 (3)	
O3	0.20598 (9)	0.93552 (15)	0.28380 (8)	0.0338 (3)	
H3	0.165707	0.899140	0.253855	0.041*	
O4	0.47133 (8)	0.05607 (13)	0.38739 (8)	0.0275 (3)	
O5A	0.25163 (15)	0.8579 (2)	0.70428 (13)	0.0338 (6)	0.637 (4)
H5A	0.283755	0.813423	0.741012	0.041*	0.637 (4)
O5B	0.3604 (2)	0.9025 (4)	0.6728 (2)	0.0282 (9)	0.363 (4)
H5B	0.358402	0.867406	0.724666	0.034*	0.363 (4)
O6A	0.08659 (10)	0.82728 (16)	0.18542 (11)	0.0337 (4)	0.8903 (18)
O6B	0.0447 (10)	0.8676 (13)	0.2106 (10)	0.044 (3)*	0.1097 (18)
N2	0.35684 (8)	0.60178 (14)	0.47516 (9)	0.0193 (3)	
C1	0.35193 (10)	0.71814 (17)	0.41916 (10)	0.0193 (3)	
C3A	0.29022 (10)	0.77925 (18)	0.55483 (10)	0.0218 (3)	
H3AA	0.232017	0.752657	0.536985	0.026*	
C3	0.34047 (11)	0.64061 (18)	0.56878 (10)	0.0231 (3)	
H3A	0.392113	0.658285	0.605867	0.028*	
H3B	0.308591	0.564525	0.597492	0.028*	
C4B	0.29054 (12)	0.8898 (2)	0.63036 (11)	0.0302 (4)	0.637 (4)
H4A	0.349158	0.910797	0.649808	0.036*	0.637 (4)
C4C	0.29054 (12)	0.8898 (2)	0.63036 (11)	0.0302 (4)	0.363 (4)
H4B	0.251734	0.853878	0.674610	0.036*	0.363 (4)
C4A	0.25370 (11)	1.02578 (19)	0.58966 (11)	0.0258 (3)	
C5A	0.19138 (11)	1.26951 (19)	0.56388 (11)	0.0264 (3)	
C6	0.15675 (13)	1.4072 (2)	0.56345 (14)	0.0338 (4)	
H6A	0.143922	1.452519	0.618055	0.041*	
C7	0.14169 (12)	1.4755 (2)	0.48199 (14)	0.0331 (4)	
H7A	0.117404	1.568665	0.480419	0.040*	
C8	0.16150 (12)	1.41029 (19)	0.40123 (13)	0.0295 (4)	
H8A	0.151707	1.460284	0.345810	0.035*	
C9B	0.24595 (10)	1.06021 (17)	0.50055 (10)	0.0204 (3)	
C9A	0.21054 (10)	1.20072 (17)	0.48381 (11)	0.0213 (3)	
C9	0.19531 (10)	1.27320 (18)	0.40184 (11)	0.0238 (3)	
H9A	0.208082	1.228670	0.346961	0.029*	
C10A	0.32805 (9)	0.84675 (16)	0.47404 (10)	0.0187 (3)	
H10A	0.380248	0.894315	0.497193	0.022*	
C10	0.27474 (9)	0.96355 (16)	0.42751 (10)	0.0184 (3)	
H10B	0.309492	1.021507	0.387981	0.022*	
C11	0.20217 (10)	0.90191 (17)	0.36979 (11)	0.0216 (3)	
C12	0.38481 (9)	0.46286 (16)	0.45148 (10)	0.0189 (3)	
C13	0.42078 (10)	0.37136 (18)	0.51699 (11)	0.0218 (3)	
H13A	0.426719	0.402232	0.577975	0.026*	
C14	0.44797 (10)	0.23566 (18)	0.49391 (11)	0.0224 (3)	
H14A	0.471177	0.173102	0.539320	0.027*	
C15	0.44140 (10)	0.19057 (16)	0.40438 (11)	0.0206 (3)	
C16	0.40420 (10)	0.28012 (17)	0.33900 (11)	0.0227 (3)	
H16A	0.398353	0.249190	0.278021	0.027*	
C17	0.37553 (10)	0.41479 (17)	0.36268 (10)	0.0214 (3)	
H17A	0.349271	0.474921	0.317799	0.026*	
C18	0.46796 (13)	0.0111 (2)	0.29506 (13)	0.0319 (4)	
H18A	0.490909	−0.086181	0.291209	0.048*	
H18B	0.410733	0.010725	0.270532	0.048*	
H18C	0.499947	0.078007	0.260286	0.048*	
C19A	0.02185 (17)	0.6061 (3)	0.0999 (2)	0.0471 (7)	0.8903 (18)
D19A	0.008139	0.503811	0.105923	0.071*	0.8903 (18)
D19B	−0.028387	0.661165	0.084572	0.071*	0.8903 (18)
D19C	0.059929	0.617892	0.052192	0.071*	0.8903 (18)
C20A	−0.01808 (16)	0.6679 (3)	0.26878 (19)	0.0470 (6)	0.8903 (18)
D20A	−0.032251	0.567942	0.282401	0.070*	0.8903 (18)
D20B	−0.005525	0.720540	0.325154	0.070*	0.8903 (18)
D20C	−0.064553	0.713967	0.234952	0.070*	0.8903 (18)
C19B	0.0423 (13)	0.6018 (18)	0.2686 (12)	0.049 (4)*	0.1097 (18)
D19D	0.010074	0.512543	0.265939	0.074*	0.1097 (18)
D19E	0.096287	0.583660	0.245646	0.074*	0.1097 (18)
D19F	0.049335	0.635024	0.331200	0.074*	0.1097 (18)
C20B	0.0041 (16)	0.666 (2)	0.0930 (9)	0.049 (4)*	0.1097 (18)
D20D	−0.029537	0.579159	0.082990	0.074*	0.1097 (18)
D20E	−0.012775	0.738825	0.047762	0.074*	0.1097 (18)
D20F	0.062047	0.641717	0.087819	0.074*	0.1097 (18)

### Atomic displacement parameters (Å^2^)

**Table:**

	*U*^11^	*U*^22^	*U*^33^	*U*^12^	*U*^13^	*U*^23^
S1A	0.0229 (2)	0.0222 (2)	0.0503 (3)	−0.00093 (17)	−0.0028 (2)	0.0081 (2)
S5	0.0489 (3)	0.0338 (2)	0.01792 (19)	0.0139 (2)	0.00149 (17)	−0.00626 (16)
O1	0.0395 (7)	0.0201 (5)	0.0168 (5)	0.0014 (5)	0.0053 (5)	−0.0001 (4)
O2	0.0332 (7)	0.0444 (8)	0.0314 (7)	−0.0135 (6)	−0.0070 (6)	0.0084 (6)
O3	0.0458 (8)	0.0352 (7)	0.0188 (6)	−0.0119 (6)	−0.0097 (5)	0.0031 (5)
O4	0.0368 (7)	0.0202 (6)	0.0260 (6)	0.0073 (5)	0.0066 (5)	0.0023 (4)
O5A	0.0472 (13)	0.0372 (12)	0.0179 (9)	0.0081 (9)	0.0094 (8)	0.0081 (8)
O5B	0.0288 (18)	0.036 (2)	0.0193 (16)	−0.0038 (14)	−0.0041 (12)	−0.0009 (13)
O6A	0.0408 (9)	0.0232 (7)	0.0346 (8)	−0.0094 (6)	−0.0158 (7)	0.0074 (6)
N2	0.0236 (6)	0.0192 (6)	0.0149 (5)	0.0026 (5)	−0.0004 (5)	0.0004 (5)
C1	0.0218 (7)	0.0183 (7)	0.0174 (6)	0.0003 (5)	−0.0009 (5)	−0.0003 (5)
C3A	0.0264 (7)	0.0258 (7)	0.0131 (6)	0.0056 (6)	0.0004 (5)	0.0014 (5)
C3	0.0288 (8)	0.0258 (8)	0.0146 (6)	0.0067 (6)	0.0007 (6)	0.0018 (6)
C4B	0.0406 (10)	0.0370 (9)	0.0127 (7)	0.0155 (8)	−0.0008 (6)	−0.0016 (6)
C4C	0.0406 (10)	0.0370 (9)	0.0127 (7)	0.0155 (8)	−0.0008 (6)	−0.0016 (6)
C4A	0.0320 (8)	0.0278 (8)	0.0172 (7)	0.0080 (7)	−0.0010 (6)	−0.0045 (6)
C5A	0.0312 (8)	0.0256 (8)	0.0222 (7)	0.0038 (7)	0.0006 (6)	−0.0037 (6)
C6	0.0391 (10)	0.0281 (9)	0.0345 (10)	0.0060 (8)	0.0054 (8)	−0.0086 (7)
C7	0.0355 (9)	0.0217 (8)	0.0424 (11)	0.0042 (7)	0.0044 (8)	−0.0021 (7)
C8	0.0324 (9)	0.0221 (8)	0.0337 (9)	0.0021 (7)	0.0005 (7)	0.0032 (7)
C9B	0.0212 (7)	0.0220 (7)	0.0179 (7)	0.0023 (6)	−0.0005 (5)	−0.0041 (5)
C9A	0.0196 (7)	0.0221 (7)	0.0218 (7)	0.0002 (5)	−0.0009 (5)	−0.0037 (6)
C9	0.0249 (7)	0.0215 (7)	0.0249 (8)	0.0006 (6)	0.0014 (6)	−0.0008 (6)
C10A	0.0212 (7)	0.0193 (7)	0.0153 (6)	0.0022 (5)	−0.0004 (5)	−0.0011 (5)
C10	0.0213 (7)	0.0192 (7)	0.0144 (6)	0.0011 (5)	−0.0015 (5)	−0.0012 (5)
C11	0.0248 (7)	0.0186 (7)	0.0205 (7)	0.0014 (6)	−0.0047 (6)	−0.0001 (5)
C12	0.0187 (6)	0.0182 (7)	0.0196 (7)	0.0002 (5)	−0.0009 (5)	0.0014 (5)
C13	0.0233 (7)	0.0239 (7)	0.0177 (7)	0.0020 (6)	−0.0023 (5)	0.0015 (6)
C14	0.0217 (7)	0.0228 (7)	0.0224 (7)	0.0036 (6)	−0.0015 (6)	0.0049 (6)
C15	0.0197 (7)	0.0176 (7)	0.0248 (7)	0.0006 (5)	0.0033 (6)	0.0018 (6)
C16	0.0289 (8)	0.0196 (7)	0.0195 (7)	−0.0010 (6)	0.0013 (6)	0.0004 (6)
C17	0.0261 (7)	0.0186 (7)	0.0188 (7)	0.0000 (6)	−0.0026 (6)	0.0018 (5)
C18	0.0453 (11)	0.0226 (8)	0.0289 (9)	0.0050 (7)	0.0111 (8)	−0.0007 (7)
C19A	0.0414 (14)	0.0399 (14)	0.0605 (17)	−0.0108 (11)	0.0066 (12)	−0.0198 (12)
C20A	0.0358 (12)	0.0560 (16)	0.0497 (15)	−0.0034 (11)	0.0075 (11)	0.0072 (12)

### Geometric parameters (Å, º)

**Table:**

S1A—O6A	1.5127 (15)	C7—C8	1.401 (3)
S1A—C20A	1.769 (3)	C7—H7A	0.9500
S1A—C19A	1.777 (3)	C8—C9	1.386 (2)
S1B—O6B	1.515 (12)	C8—H8A	0.9500
S1B—C19B	1.762 (12)	C9B—C9A	1.441 (2)
S1B—C20B	1.774 (13)	C9B—C10	1.507 (2)
S5—C4A	1.7407 (17)	C9A—C9	1.400 (2)
S5—C5A	1.7434 (18)	C9—H9A	0.9500
O1—C1	1.2288 (19)	C10A—C10	1.526 (2)
O2—C11	1.207 (2)	C10A—H10A	1.0000
O3—C11	1.322 (2)	C10—C11	1.527 (2)
O3—H3	0.8400	C10—H10B	1.0000
O4—C15	1.3692 (19)	C12—C13	1.392 (2)
O4—C18	1.433 (2)	C12—C17	1.393 (2)
O5A—C4B	1.338 (3)	C13—C14	1.385 (2)
O5A—H5A	0.8400	C13—H13A	0.9500
O5B—C4C	1.271 (4)	C14—C15	1.393 (2)
O5B—H5B	0.8400	C14—H14A	0.9500
N2—C1	1.362 (2)	C15—C16	1.388 (2)
N2—C12	1.419 (2)	C16—C17	1.387 (2)
N2—C3	1.479 (2)	C16—H16A	0.9500
C1—C10A	1.511 (2)	C17—H17A	0.9500
C3A—C4C	1.521 (2)	C18—H18A	0.9800
C3A—C4B	1.521 (2)	C18—H18B	0.9800
C3A—C10A	1.521 (2)	C18—H18C	0.9800
C3A—C3	1.532 (2)	C19A—D19A	0.9800
C3A—H3AA	1.0000	C19A—D19B	0.9800
C3—H3A	0.9900	C19A—D19C	0.9800
C3—H3B	0.9900	C20A—D20A	0.9800
C4B—C4A	1.507 (2)	C20A—D20B	0.9800
C4B—H4A	1.0000	C20A—D20C	0.9800
C4C—C4A	1.507 (2)	C19B—D19D	0.9800
C4C—H4B	1.0000	C19B—D19E	0.9800
C4A—C9B	1.361 (2)	C19B—D19F	0.9800
C5A—C6	1.396 (3)	C20B—D20D	0.9800
C5A—C9A	1.404 (2)	C20B—D20E	0.9800
C6—C7	1.375 (3)	C20B—D20F	0.9800
C6—H6A	0.9500		
			
O6A—S1A—C20A	106.26 (13)	C8—C9—C9A	119.61 (16)
O6A—S1A—C19A	104.20 (12)	C8—C9—H9A	120.2
C20A—S1A—C19A	99.03 (13)	C9A—C9—H9A	120.2
O6B—S1B—C19B	105.6 (8)	C1—C10A—C3A	103.55 (12)
O6B—S1B—C20B	105.2 (8)	C1—C10A—C10	118.36 (12)
C19B—S1B—C20B	100.2 (9)	C3A—C10A—C10	113.66 (13)
C4A—S5—C5A	91.61 (8)	C1—C10A—H10A	106.9
C11—O3—H3	109.5	C3A—C10A—H10A	106.9
C15—O4—C18	116.77 (13)	C10—C10A—H10A	106.9
C4B—O5A—H5A	109.5	C9B—C10—C10A	106.90 (12)
C4C—O5B—H5B	109.5	C9B—C10—C11	111.15 (13)
C1—N2—C12	125.06 (13)	C10A—C10—C11	112.76 (13)
C1—N2—C3	112.06 (13)	C9B—C10—H10B	108.6
C12—N2—C3	122.38 (13)	C10A—C10—H10B	108.6
O1—C1—N2	127.11 (15)	C11—C10—H10B	108.6
O1—C1—C10A	125.25 (14)	O2—C11—O3	124.33 (15)
N2—C1—C10A	107.58 (13)	O2—C11—C10	123.87 (15)
C4C—C3A—C10A	108.91 (14)	O3—C11—C10	111.81 (14)
C4B—C3A—C10A	108.91 (14)	C13—C12—C17	118.87 (14)
C4C—C3A—C3	119.35 (13)	C13—C12—N2	120.50 (14)
C4B—C3A—C3	119.35 (13)	C17—C12—N2	120.62 (13)
C10A—C3A—C3	102.18 (12)	C14—C13—C12	120.51 (15)
C4B—C3A—H3AA	108.6	C14—C13—H13A	119.7
C10A—C3A—H3AA	108.6	C12—C13—H13A	119.7
C3—C3A—H3AA	108.6	C13—C14—C15	120.25 (14)
N2—C3—C3A	101.83 (12)	C13—C14—H14A	119.9
N2—C3—H3A	111.4	C15—C14—H14A	119.9
C3A—C3—H3A	111.4	O4—C15—C16	124.11 (15)
N2—C3—H3B	111.4	O4—C15—C14	116.36 (14)
C3A—C3—H3B	111.4	C16—C15—C14	119.51 (14)
H3A—C3—H3B	109.3	C17—C16—C15	120.02 (15)
O5A—C4B—C4A	108.46 (16)	C17—C16—H16A	120.0
O5A—C4B—C3A	118.61 (19)	C15—C16—H16A	120.0
C4A—C4B—C3A	106.60 (13)	C16—C17—C12	120.76 (14)
O5A—C4B—H4A	107.6	C16—C17—H17A	119.6
C4A—C4B—H4A	107.6	C12—C17—H17A	119.6
C3A—C4B—H4A	107.6	O4—C18—H18A	109.5
O5B—C4C—C4A	116.3 (2)	O4—C18—H18B	109.5
O5B—C4C—C3A	112.9 (2)	H18A—C18—H18B	109.5
C4A—C4C—C3A	106.60 (13)	O4—C18—H18C	109.5
O5B—C4C—H4B	106.9	H18A—C18—H18C	109.5
C4A—C4C—H4B	106.9	H18B—C18—H18C	109.5
C3A—C4C—H4B	106.9	S1A—C19A—D19A	109.5
C9B—C4A—C4C	126.63 (15)	S1A—C19A—D19B	109.5
C9B—C4A—C4B	126.63 (15)	D19A—C19A—D19B	109.5
C9B—C4A—S5	112.36 (13)	S1A—C19A—D19C	109.5
C4C—C4A—S5	120.99 (12)	D19A—C19A—D19C	109.5
C4B—C4A—S5	120.99 (12)	D19B—C19A—D19C	109.5
C6—C5A—C9A	121.61 (17)	S1A—C20A—D20A	109.5
C6—C5A—S5	127.31 (14)	S1A—C20A—D20B	109.5
C9A—C5A—S5	111.07 (13)	D20A—C20A—D20B	109.5
C7—C6—C5A	118.33 (17)	S1A—C20A—D20C	109.5
C7—C6—H6A	120.8	D20A—C20A—D20C	109.5
C5A—C6—H6A	120.8	D20B—C20A—D20C	109.5
C6—C7—C8	121.20 (17)	S1B—C19B—D19D	109.5
C6—C7—H7A	119.4	S1B—C19B—D19E	109.5
C8—C7—H7A	119.4	D19D—C19B—D19E	109.5
C9—C8—C7	120.31 (17)	S1B—C19B—D19F	109.5
C9—C8—H8A	119.8	D19D—C19B—D19F	109.5
C7—C8—H8A	119.8	D19E—C19B—D19F	109.5
C4A—C9B—C9A	113.02 (14)	S1B—C20B—D20D	109.5
C4A—C9B—C10	123.29 (14)	S1B—C20B—D20E	109.5
C9A—C9B—C10	123.65 (14)	D20D—C20B—D20E	109.5
C9—C9A—C5A	118.92 (15)	S1B—C20B—D20F	109.5
C9—C9A—C9B	129.13 (15)	D20D—C20B—D20F	109.5
C5A—C9A—C9B	111.94 (15)	D20E—C20B—D20F	109.5
			
C12—N2—C1—O1	3.3 (3)	C10—C9B—C9A—C9	0.6 (3)
C3—N2—C1—O1	175.30 (16)	C4A—C9B—C9A—C5A	−0.6 (2)
C12—N2—C1—C10A	−174.00 (13)	C10—C9B—C9A—C5A	−178.18 (15)
C3—N2—C1—C10A	−1.96 (18)	C7—C8—C9—C9A	−0.8 (3)
C1—N2—C3—C3A	23.14 (17)	C5A—C9A—C9—C8	−0.3 (2)
C12—N2—C3—C3A	−164.58 (14)	C9B—C9A—C9—C8	−179.02 (17)
C4C—C3A—C3—N2	−154.00 (15)	O1—C1—C10A—C3A	162.34 (16)
C4B—C3A—C3—N2	−154.00 (15)	N2—C1—C10A—C3A	−20.34 (16)
C10A—C3A—C3—N2	−33.89 (15)	O1—C1—C10A—C10	35.5 (2)
C10A—C3A—C4B—O5A	172.66 (17)	N2—C1—C10A—C10	−147.14 (14)
C3—C3A—C4B—O5A	−70.7 (2)	C4C—C3A—C10A—C1	160.51 (13)
C10A—C3A—C4B—C4A	50.03 (19)	C4B—C3A—C10A—C1	160.51 (13)
C3—C3A—C4B—C4A	166.67 (15)	C3—C3A—C10A—C1	33.35 (15)
C10A—C3A—C4C—O5B	−78.8 (2)	C4C—C3A—C10A—C10	−69.78 (17)
C3—C3A—C4C—O5B	37.8 (3)	C4B—C3A—C10A—C10	−69.78 (17)
C10A—C3A—C4C—C4A	50.03 (19)	C3—C3A—C10A—C10	163.06 (13)
C3—C3A—C4C—C4A	166.67 (15)	C4A—C9B—C10—C10A	−12.4 (2)
O5B—C4C—C4A—C9B	107.8 (3)	C9A—C9B—C10—C10A	165.01 (14)
C3A—C4C—C4A—C9B	−19.0 (3)	C4A—C9B—C10—C11	111.08 (18)
O5B—C4C—C4A—S5	−70.3 (3)	C9A—C9B—C10—C11	−71.54 (19)
C3A—C4C—C4A—S5	162.82 (13)	C1—C10A—C10—C9B	168.32 (13)
O5A—C4B—C4A—C9B	−147.8 (2)	C3A—C10A—C10—C9B	46.52 (17)
C3A—C4B—C4A—C9B	−19.0 (3)	C1—C10A—C10—C11	45.88 (19)
O5A—C4B—C4A—S5	34.0 (2)	C3A—C10A—C10—C11	−75.93 (17)
C3A—C4B—C4A—S5	162.82 (13)	C9B—C10—C11—O2	−57.9 (2)
C5A—S5—C4A—C9B	0.01 (15)	C10A—C10—C11—O2	62.1 (2)
C5A—S5—C4A—C4C	178.43 (16)	C9B—C10—C11—O3	122.07 (15)
C5A—S5—C4A—C4B	178.43 (16)	C10A—C10—C11—O3	−117.90 (15)
C4A—S5—C5A—C6	−179.43 (19)	C1—N2—C12—C13	152.25 (16)
C4A—S5—C5A—C9A	−0.33 (14)	C3—N2—C12—C13	−19.0 (2)
C9A—C5A—C6—C7	−0.2 (3)	C1—N2—C12—C17	−28.8 (2)
S5—C5A—C6—C7	178.84 (16)	C3—N2—C12—C17	159.92 (15)
C5A—C6—C7—C8	−0.9 (3)	C17—C12—C13—C14	1.0 (2)
C6—C7—C8—C9	1.4 (3)	N2—C12—C13—C14	179.89 (15)
C4C—C4A—C9B—C9A	−178.00 (17)	C12—C13—C14—C15	1.6 (2)
C4B—C4A—C9B—C9A	−178.00 (17)	C18—O4—C15—C16	4.1 (2)
S5—C4A—C9B—C9A	0.3 (2)	C18—O4—C15—C14	−177.49 (15)
C4C—C4A—C9B—C10	−0.4 (3)	C13—C14—C15—O4	178.70 (15)
C4B—C4A—C9B—C10	−0.4 (3)	C13—C14—C15—C16	−2.8 (2)
S5—C4A—C9B—C10	177.93 (13)	O4—C15—C16—C17	179.86 (15)
C6—C5A—C9A—C9	0.8 (3)	C14—C15—C16—C17	1.5 (2)
S5—C5A—C9A—C9	−178.38 (13)	C15—C16—C17—C12	1.1 (2)
C6—C5A—C9A—C9B	179.71 (17)	C13—C12—C17—C16	−2.3 (2)
S5—C5A—C9A—C9B	0.55 (19)	N2—C12—C17—C16	178.78 (15)
C4A—C9B—C9A—C9	178.24 (17)		

### Hydrogen-bond geometry (Å, º)

**Table:**

*D*—H···*A*	*D*—H	H···*A*	*D*···*A*	*D*—H···*A*
O3—H3···S1*A*	0.84	2.72	3.4846 (15)	152
O3—H3···O6*A*	0.84	1.72	2.563 (2)	176
O3—H3···O6*B*	0.84	2.06	2.852 (16)	158
O5*A*—H5*A*···O1^i^	0.84	1.96	2.763 (2)	160
C3*A*—H3*AA*···O2	1.00	2.49	3.202 (2)	127
C3—H3*A*···O5*B*	0.99	2.54	2.887 (4)	100
C6—H6*A*···O6*A*^ii^	0.95	2.48	3.307 (3)	145
C14—H14*A*···O4^iii^	0.95	2.54	3.445 (2)	159
C17—H17*A*···O1	0.95	2.33	2.868 (2)	116
C17—H17*A*···O5*B*^iv^	0.95	2.46	3.289 (4)	146
C18—H18*C*···O5*B*^v^	0.98	2.43	2.922 (4)	110
C20*A*—D20*A*···O6*A*^vi^	0.98	2.46	3.434 (3)	173
C20*A*—D20*A*···O6*B*^vi^	0.98	1.87	2.839 (13)	168

Symmetry codes: (i) *x*, −*y*+3/2, *z*+1/2; (ii) *x*, −*y*+5/2, *z*+1/2; (iii) −*x*+1, −*y*, −*z*+1; (iv) *x*, −*y*+3/2, *z*−1/2; (v) −*x*+1, −*y*+1, −*z*+1; (vi) −*x*, *y*−1/2, −*z*+1/2.
